# Supplementary material for: The Gut Microbiome, Aging, and Longevity: A Systematic Review
Source: Nutrients. 2020 Dec 7;12(12):3759. doi: 10.3390/nu12123759 (PMC7762384; doi:10.3390/nu12123759)
Supplement: Supplementary file 1 [file nutrients-12-03759-s001.pdf]

Supplementary Material

Table S1. Sample and clinical characteristics of reviewed studies

Publication	Country	Rural/Urban	Sample Sizes	Mean Age	Gender	Sample Characteristics	Clinical Assessments
Centenarian Studies							
Biagi et al., 2016	Italy	Urban	SCTN: 24<br>LL: 15<br>YO: 15<br>ADT: 15	SCTN: 106.2 (SD=1.4; range 105-109)<br>LL: 100.4 (SD=1.4; range 99-104)<br>YO: 72.5 (SD=3.7; range 65-75)<br>ADT: 30.5 (SD=7.9; range 22-48)	SCTN: 6M:18F<br>LL: 1M:14F<br>YO: 8M:7F<br>ADT: 7M:8F	SCTN: 7 in nursing homes, 19 severely disabled, 5 affected by cancer in the past, 3 with cancer at the time of assessment; on variety of medications; BMI for 13 participants: 23.2 (SD=4.2)<br>SCTN and LL: Physical and cognitive health status matched the majority of Italian centenarians<br>YO: from the same regions as SCTN; no info on BMI<br>ADT: recruited from the same regions as SCTN; healthy; medication-free; followed Mediterranean diet; no info on BMI	None reported
Drago et al., 2012	Italy	Urban	CTN: 14<br>ADT: 10	CTN: not reported (range 100-104)<br>ADT: not reported (range 24-57)	CTN: not reported<br>ADT: not reported	CTN: recruited from nursing homes; no history of chronic inflammatory bowel diseases or metabolic diseases; no antibiotics or probiotics one month before sampling period; no info on BMI<br>ADT: no history of chronic inflammatory bowel diseases or metabolic diseases; no antibiotics or probiotics one month before sampling period; no info on BMI	None reported
Kim et al., 2019	Republic of South Korea	Rural	LL: 30<br>YO: 17<br>ADT: 9	LL: 98.9 (SD=3.4; range 95-108)<br>YO: 73.6 (SD=3.6; range 67-79)	LL: 3M:27F<br>YO: 10M:7F<br>ADT: 6M:3F	LL: 20 community-dwelling; 10 in rehabilitation hospitals; all from longevity villages; no systemic antibiotic use within one month prior to sampling; no info on BMI<br>YO: community-dwelling; recruited from same regions (urbanized towns or longevity villages); no info on BMI	None reported

				ADT: 34.3 (SD=6.5; range 26-43)		ADT: community-dwelling; recruited from same regions (urbanized towns or longevity villages); no info on BMI	
Kong et al., 2016	China	Urban	LL: 67<br><br>ADT: 101	LL: 93.3 (SD=3.1; range 90-102)<br><br>ADT: 62.0 (SD=14.1; range: 24-83)	LL: 26M:41F<br><br>ADT: 52M:44F	LL: community-dwelling; no info on BMI<br><br>ADT: recruited from the same regions as LL; no info on BMI	None reported
Rampelli et al., 2013	Italy	Urban	LL: 3<br><br>YO: 5<br><br>ADT: 1	LL: 100.7 (SD=not reported; range 99-102)<br><br>YO: 66.4 (SD=not reported; range 59-75)<br><br>ADT: 38	LL: not reported<br><br>YO: not reported<br><br>ADT: not reported	LL: recruited from region in Northern Italy; no info on BMI<br><br>YO: recruited from the same regions as LL; 3 were offspring of LL; no info on BMI<br><br>ADT: recruited from same region as LL; no info on BMI<br><br>All subjects were overlapping with Biagi et al., 2016.	None reported
Tuikhar et al., 2019	India	Rural	LL: 30<br><br>ADT internal: 30<br><br>ADT external: 30	LL: 99.9 (SD=3.55; range 97-110)<br><br>ADT internal: 35.8 (SD=6.3; range 28-47)<br><br>ADT external: 34.8 (SD=7.9; range 22-50)	LL: 15M:15F<br><br>ADT internal: 12M:18F<br><br>ADT external: 15M:15F	LL: Chandel and Senapati districts of Manipur state (high centenarian prevalence); no antibiotic treatment within 6 months prior of sampling; no info on BMI<br><br>ADT internal: recruited from same regions as LL; no antibiotic treatment within 6 months prior of sampling; no info on BMI<br><br>ADT external: recruited from different regions from LL with low centenarian prevalence (Meitei community of Imphal West district); no antibiotic treatment within 6 months prior of sampling; no info on BMI	None reported
Wu et al., 2019	Italy	Rural	LL: 19<br><br>YO: 23	LL: 101.8 (SD=2.0; range 99-107)	LL: 6M:13F<br><br>YO: 10M:13F	LL: no history of chronic medical conditions; no antimicrobial medication 1 year prior to sampling; BMI: 23.5 (SD=2.8)	None reported

			ADT: 17	YO: 77.2 (SD=6.7; range 68-88)<br><br>ADT: 25.5 (SD=4.0; range 21-33)	ADT: 7M:10F	YO: no history of chronic medical conditions; no antimicrobial medication 1 year prior to sampling; BMI: 25.9 (SD=5.2)<br><br>ADT: no history of chronic medical conditions; no antimicrobial medication 1 year prior to sampling; BMI: 22.8 (SD=5.5)	
Yu et al., 2015	China	Rural	LL: 21<br><br>CK: 28	LL: 73.2 (SD=12.7; range 50-95)<br><br>CK: 50 (SD=not reported; range not reported)	LL: 10M:11F<br><br>CK: Not reported	LL: no antibiotic use 6 months prior to sampling; no hx of gastrointestinal diseases; from Gaotian longevity villages; no info on BMI<br><br>CK: control group; no antibiotic use 6 months prior to sampling; no hx of gastrointestinal diseases; no long-living record in their families; no info on BMI	None reported
Lifespan Studies							
Claesson et al. 2012	Ireland	Urban	YO: 178<br><br>ADT: 13	YO: 78 (SD=8; 64-102)<br><br>ADT: 36 (SD=6; range 28-46)	YO: not reported<br><br>ADT: not reported	YO: 83 community-dwelling; 20 day-hospital; 15 short-term rehabilitation hospital care; 60 long-term care facilities; no hx of alcohol abuse or advanced organic disease; no antibiotic treatments or participation in investigational drug evaluation within 30 days prior to sampling; no info on BMI<br><br>ADT: control group; no info on matching; no antibiotic treatment within 30 days prior to sampling; no info on BMI	Diet (FFQ, MNA)
Hippe et al., 2011	Austria	Urban	YO: 15<br><br>ADT vegetarians: 15<br><br>ADT omnivores: 17	YO: 86 (SD=8; range not reported)<br><br>ADT vegetarians: 26 (SD=5; range not reported)<br><br>ADT omnivores: 24 (SD=2.5; range not reported)	YO: not reported<br><br>ADT vegetarians: not reported<br><br>ADT omnivores: not reported	YO: institutionalized; BMI: 21.8 (SD=5.1),<br><br>ADT vegetarians: BMI 21.0 (SD=2.7)<br><br>ADT omnivores: BMI 22.7 (SD= 3.4)	None reported

Hopkins et al. 2002	United Kingdom	Urban	NHYO: 4<br><br>YO: 5<br><br>ADT: 7<br><br>CHD: 10	NHYO: not reported (range 68-73)<br><br>YO: not reported (range 67-88)<br><br>ADT: not reported (range 21-34)<br><br>CHD: not reported (range 16 months-7)	NHYOL: not reported<br><br>YO: not reported<br><br>ADT: not reported<br><br>CHD: not reported	NHYO: diagnosed with CDAD at the time of sampling; no info on BMI<br><br>YO: no history of gastrointestinal disease; no antibiotic use within 2 months prior to sampling<br><br>ADT: no history of gastrointestinal disease; no antibiotic use within 2 months prior to sampling<br><br>CHD: no history of gastrointestinal disease; no antibiotic use within 2 months prior to sampling	None reported
Jeffery et al., 2016	Ireland	Urban	YO: 371<br><br>ADT: 13	YO: 78 (SD=8; range 64-102)<br><br>ADT: 36 (SD=6; range 28-46)	YO: not reported<br><br>ADT: not reported	YO: 179 community-dwelling, 44 day-hospital, 41 in short-term rehabilitation care,107 in long-term care facilities; no hx of: alcohol abuse, participation in drug intervention, advanced organic disease; no info on BMI<br><br>ADT: no antibiotic treatment within 30 days prior to sampling; no info on BMI	None reported
Kato et al., 2017	Japan	Urban	Age Groups<br>100: 5<br><br>90: 19<br><br>80: 51<br><br>70: 31<br><br>60: 42<br><br>50: 29<br><br>40: 37<br><br>30: 114	100: 101.6 (SD=1.8; range: 100 and up)<br><br>90: 94.2 (SD=2.7; range 90-99)<br><br>80: 83.2 (SD=2.4; range 80-89)<br><br>70: 75.5 (SD=2.9; range 70-79)	100: 0M:5F<br><br>90: 4M:15F<br><br>80: 17M:34F<br><br>70: 12M:19F<br><br>60: 14M:28F<br><br>50: 13M:16F<br><br>40: 13M:24F<br><br>30: 54M:60F	All subjects were community-dwelling	None reported

			20: 42<br>10: 10<br>4: 17<br>3: 21<br>2: 12<br>1: 16	60: 64.2 (SD=2.9; range 60-69)<br>50: 53.5 (SD=2.7; range 50-59)<br>40: 43.7 (SD=3.1; range 40-49)<br>30: 34.3 (SD=2.5; range 30-39)<br>20: 25.8 (SD=2.7; range 20-29)<br>10: 14.1 (SD=3.6; range 10-19)<br>4: 5.9 (SD=1.8; range 4-9)<br>3: 2.4 (SD=0.6; range Weaned-3 years old)<br>2: 0.8 (SD=0.4; Weaning)<br>1: 0.3 (SD=0.1; preweaning)	20: 16M:26F<br>10: 7M:3F<br>4: 7M:10F<br>3: 10M:11F<br>2: 7M:5F<br>1: 9M:7F		
Kushugulova et al., 2015	Kazakhstan	Not reported	LL: 6<br>YO: 17<br>ADT: 6	LL: not reported (range 90 and up)	LL: 0M:6F<br>YO: 0M:17F<br>ADT: 0M:6F	LL: healthy; no info on BMI<br>YO: healthy; no info on BMI<br>ADT: healthy; no info on BMI	None reported

				YO: not reported (range 50-70)<br><br>ADT: not reported (range 30-44)			
Le Roy et al., 2015	Estonia	Urban	YO: 33<br><br>ADT: 16	YO: not reported (range 65-81)<br><br>ADT: not reported (range 20-48)	YO: not reported<br><br>ADT: not reported	YO: generally healthy; followed a standard Western-type diet; no info on BMI<br><br>ADT: healthy; followed a standard Western-type diet; no info on BMI	None reported
Odamaki et al., 2016	Japan	Not reported	Age Groups<br>100: 6<br><br>90: 19<br><br>80: 48<br><br>70: 15<br><br>60: 28<br><br>50: 25<br><br>40: 34<br><br>30: 88<br><br>20: 40<br><br>10: 10<br><br>4: 14<br><br>3: 18<br><br>2: 12<br><br>1: 14	100: 101.3 (SD=1.8; range: 100 and up)<br><br>90: 94.2 (SD=2.7; range 90-99)<br><br>80: 83.3 (SD=2.4; range 80-89)<br><br>70: 76.8 (SD=2.1; range 70-79)<br><br>60: 63 (SD=2.7; range 60-69)<br><br>50: 53.3 (SD=2.6; range 50-59)<br><br>40: 43.8 (SD=3.1; range 40-49)	100: 0M:6F<br><br>90: 4M:15F<br><br>80: 16M:32F<br><br>70: 5M:10F<br><br>60: 11M:17F<br><br>50: 12M:13F<br><br>40: 13M:21F<br><br>30: 45M:43F<br><br>20: 16M:24F<br><br>10: 7M:3F<br><br>4: 6M:8F<br><br>3: 10M:8F<br><br>2: 6M:6F<br><br>1: 7M:7F	All participants were community-dwelling; no info on BMI	None reported

				30: 33.9 (SD=2.3; range 30-39)<br><br>20: 25.9 (SD=2.7; range 20-29)<br><br>10: 14.1 (SD=3.6; range 10-19)<br><br>4: 6.1 (SD=1.9; range 4-9)<br><br>3: 2.4 (SD=0.6; range Weaned-3 years old)<br><br>2: 0.8 (SD=0.4; Weaning)<br><br>1: 0.3 (SD=0.1; preweaning)			
Odamaki et al., 2018	Japan	Urban	Age Groups<br>100: 6<br><br>90: 19<br><br>80: 51<br><br>70: 31<br><br>60: 42<br><br>50: 34<br><br>40: 37<br><br>30: 117<br><br>20: 42	100: 101.3 (SD=1.8; range: 100 and up)<br><br>90: 94.2 (SD=2.7; range 90-99)<br><br>80: 83.2 (SD=2.4; range 80-89)<br><br>70: 75.5 (SD=2.9; range 70-79)<br><br>60: 64.2 (SD=2.9; range 60-69)	100: 0M:6F<br><br>90: 4M:15F<br><br>80: 17M:34F<br><br>70: 12M:19F<br><br>60: 14M:28F<br><br>50: 14M:20F<br><br>40: 14M:23F<br><br>30: 56M:61F<br><br>20: 14M:28F<br><br>10: 7M:3F	All subjects were community-dwelling; no info on BMI	None reported

			10: 10<br>4: 17<br>3: 22<br>2: 12<br>1: 13	50: 53.7 (SD=2.8; range 50-59)<br>40: 43.4 (SD=3.1; range 40-49)<br>30: 34.3 (SD=2.6; range 30-39)<br>20: 25.8 (SD=2.6; range 20-29)<br>10: 14.1 (SD=3.6; range 10-19)<br>4: 5.9 (SD=1.8; range 4-9)<br>3: 2.4 (SD=0.6; range Weaned-3 years old)<br>2: 0.8 (SD=0.4; Weaning)<br>1: 0.3 (SD=0.1; preweaning)	4: 7M:10F<br>3: 11M:11F<br>2: 8M:4F<br>1: 7M:6F		
Pan et al., 2016	China	Rural	LL Bama: 8<br>LL Nanning: 8	LL Bama: 92 (SD not reported; range 80-99)<br>LL Nanning: 82.8 (SD not reported; range 80-99)	LL Bama: 3M:5F<br>LL Nanning: 4M:4F	LL Bama: from Bama (longevity region); healthy; no antibiotic use within 1 month prior to sampling; no info on BMI<br>LL Nanning: from Nanning (not considered a longevity region); healthy; no antibiotic use within 1 month prior to sampling; no info on BMI	Dietary information (FFQ 23 and China Food Composition)

Ruiz-Ruiz et al., 2019	Spain	Urban	YO: 10<br><br>ADT: 10<br><br>CHD: 10	YO: 74.5 (SD=4.2; range 68-81)<br><br>ADT: 35.4 (SD=6.6; range 27-44)<br><br>CHD: 3.9 (SD=1.4; range 2-5)	YO: 3M:7F<br><br>ADT: 5M:5F<br><br>CHD: 5M:5F	YO: no history of intestinal organic disorders, chronic diseases; no recent antibiotic treatment; no info on BMI<br><br>ADT: no history of intestinal organic disorders, chronic diseases; no recent antibiotic treatment; no info on BMI<br><br>CHD: no history of intestinal organic disorders, chronic diseases; no recent antibiotic treatment; no info on BMI	None reported
Singh et al., 2019	USA	Rural	YO: 33<br><br>NHYO: 32	YO: 75.5 (SD=5.7; range 70-82)<br><br>NHYO: 72.7 (SD=3.5; range 70-82)	YO: 14M:19F<br><br>NHYO: 17M:15F	YO: no reported diagnosis of major diseases<br><br>NHYO: medical hx of CA, CVD, PD, CLD, DM, stroke, or ND	None reported
						Cognition Studies	
Anderson et al., 2017	USA	Not reported	YO: 37	YO: 64.6 (SD=7.5; range 50-85)	YO: 10M:27F	YO: community-dwelling; no history of neurological, developmental, or severe psychiatric disorder; no antibiotic or probiotic use within 30 days prior to sampling; no hx of significant gastrointestinal disorder/surgery; no history of alcohol or illicit drug dependence; no history of severe heart, kidney, or liver problems; no info on BMI	Cognitive flexibility (SCWT); Sleep quality (PSQI)
Manderino et al., 2017	USA	Urban	YO: 25<br><br>NHYO: 18	YO: 64.1 (SD=6.5; range 50-85)<br><br>NHYO: 64.1 (SD=9.4; range 50-85)	YO: 17M:8F<br><br>NHYO: 12M:6F	YO: ≤1 impaired score; no info on BMI<br><br>NHYO: Performed ≥1 SD below normative performance on two or more tests; no info on BMI<br><br>All had no hx of neurological, developmental, or severe psychiatric disorder; no antibiotic or probiotic use within 30 days prior to sampling; no history of significant gastrointestinal disorder or surgery; no history of alcohol or illicit drug dependence;	Cognitive function (NTB); Global cognitive function (MMSE); attention function (FAB, TMT-A, TMT-B, SCWT); memory (HVLT-R, RCFT); language (FAS)

						no history of severe heart, kidney, or liver problems	
Verdi et al., 2018	United Kingdom	Urban/Rural	YO: 1551	YO: 63 (SD not reported; range 40-89)	YO: 515M:1036F	YO: from TwinsUK British cohort; community-dwelling; no current diagnosis of dementia, BMI: 25.8 (SD=4.7) for 1368 of 1551	Reaction time (DLRT); verbal fluency (Addenbrookes Cognitive Examination III); frailty (Fried phenotype); cognitive function (CANTAB-PAL); mental health (MMSE)
Intervention Studies							
An et al., 2019	Netherlands	Urban	YO pectin: 24<br><br>YO placebo: 24<br><br>ADT pectin: 25<br><br>ADT placebo: 27	YO pectin: 69.5 (SD=3.1; range 65-75)<br><br>YO placebo: 69.8 (SD=2.4; range 65-75)<br><br>ADT pectin: 23.4 (SD=4.5; range 18-40)<br><br>ADT placebo: 22.8 (SD=4.1; range 18-40)	YO pectin: 15M/9F<br><br>YO placebo: 12M:12F<br><br>ADT pectin: 8M/17F<br><br>ADT placebo: 14M/13F	YO pectin: given pectin extracted from sugar beet pulp; BMI: 25.5 (SD=2.6)<br><br>YO placebo: given maltodextrin; BMI: 26.2 (SD=2.8)<br><br>ADT pectin: given pectin extracted from sugar beet pulp; BMI: 23.2 (SD=2.7)<br><br>ADT placebo: given maltodextrin; BMI: 22.6 (SD=2.7)<br><br>All participants did/were not: have GI diseases, abdominal surgery, use anti-inflammatory drugs, and/or vitamin supplementation within 14 days prior to sampling; use pro-, pre-, or antibiotics in the 90 days prior to the study; pregnant or lactating; smoking; have hx of side effects toward prebiotic supplements; all participants took the supplements twice a day (15g/days)	None reported
Björklund et al., 2011	Finland	Urban	YO synbiotic: 23<br><br>YO placebo: 24	YO synbiotic: 71.7 (SD=6.2; range above 65)<br><br>YO placebo: 70.3 (SD=7.2;	YO synbiotic: 5M:19F<br><br>YO placebo: 7M:16F	YO synbiotic: given synbiotic product comprising of a combination of lactitol and *L. acidophilus* NCFM group; regular use of NSAID (≥3 times per week); no antibiotic use within one month prior to sampling; no info on BMI	None reported

				range above 65)		YO placebo: given saccharose; regular use of NSAID (≥3 times per week); no critical illness; no antibiotic use within one month prior to sampling; no info on BMI<br><br>All took capsules twice a day.	
Spaiser et al., 2015	USA	Urban	YO probiotic: 16<br><br>YO placebo: 16	YO probiotic: not reported<br><br>YO placebo: not reported<br><br>YO total: 69.8 (SD=0.7; range 65-80)	YO probiotic: not reported<br><br>YO placebo: not reported<br><br>YO total: 10M:22F	YO probiotic: given capsules containing mixture of *Lactobacillus gasseri* KS-13, *Bifidobacterium bifidum* G9-1, *Bifidobacterium longum* MM2 (1.5 billion viable cells per capsule), potato starch, and silicon dioxide<br><br>YO placebo: given capsules containing potato starch and silicon dioxide<br><br>YO total: capsules were taken twice a day; no immune-enhancing dietary supplements, unpasteurized fermented foods, or non-study probiotics; no antibiotic use within 2 months prior to sampling; non-smokers; no gastrointestinal disease; no chemotherapy within a year of sampling; BMI: 28.0 (SD=0.9)	None reported
Valentini et al., 2014	France, Germany, and Italy	Urban	YO with diet and VSL#3 treatment: 31<br><br>YO with diet alone: 31	YO total: 70.1 (SD=3.9; range 65-85)<br><br>Not reported for each treatment arm	YO total: 29M:33F (not reported for each treatment arm)	YO with diet and VSL#3 treatment: given capsules (taken twice daily for 8 weeks) containing 112 billion lyophilized bacteria that included *Bifidobacterium infantis* DSM 24737, *Bifidobacterium longum* DSM 24736, *Bifidobacterium breve* DSM 24732, *Lactobacillus acidophilus* DSM 24735, *Lactobacillus delbrückii* ssp. bulgaricus DSM 24734, *Lactobacillus paracasei* DSM 24733, *Lactobacillus plantarum* DSM 24730, and *Streptococcus thermophilus* DSM 2473, microcrystalline cellulose, stearic acid, magnesium stearate, silicon dioxide and coloring agent; followed RISTOMED diet plan for 8 weeks<br><br>YO with diet alone: followed RISTOMED diet plan for 8 weeks<br><br>All had no chronic severe diseases; cancers; gastrointestinal diseases requiring treatment;	Inflammation (hsCRP, ESR, WBC, fibrinogen, IL-6, TNF-a, IL-10)<br><br>No inflammation subgroup defined by hsCRP < 3 mg/l<br><br>Low-grade inflammation subgroup defined by hsCRP ≥ 3 mg/l

	DM; no current infection and antibiotic treatment, no anti-inflammatory drugs within 4 months prior to sampling; no intake of conventional yoghurt, probiotics, prebiotics and symbiotics or other supposed functional foods within the 3 weeks prior to sampling; BMI for full sample: 26.8 (SD=3.6)

Abbreviations Used: ADT: adult; BMI: body mass index; CA: cancer; CANTAB-PAL: Cambridge Neuropsychological Test Automated Battery-Paired-Associated Learning Test; CDAD: C. difficile-associated diarrhea; CHD: children; CK: control group; CLD: chronic liver disease; CRP: C-reactive protein; CTN: centenarian group; CVD: cardiovascular disease; DLRT: Deary-Liewald Reaction Time; DM: diabetes; ESR: erythrocyte sedimentation rate; F: female; FAB: Frontal Assessment Battery; FAS: Verbal Association Fluency; FFQ: Food Frequency Questionnaire; FMT: fecal microbiota transplantation; G60: ≥60 years; GI: gastrointestinal; hs-CRP: high sensitivity C-reactive protein; HVLT-R: Hopkins Verbal Learning Test – Revised; hx: history; L60: <60 years; LL: long-living, oldest-old adults; M: male; MMSE: Mini-Mental State Examination; MNA: mini nutritional assessment; ND: neurodegenerative disease; NHYO: non-healthy young-old adult; NSAID: nonsteroidal anti-inflammatory drugs; NTB: neuropsychological test battery; PD: pulmonary disease; PSQI: Pittsburgh Sleep Quality Index; RCDI: recurrent Clostridium difficile infection; RCFT: Rey-Osterrieth Complex Figure Task; SCTN: semi-supercentenarians; SCWT: Stroop Color Word Test; TMT-A: Trail Making Test A; TMT-B: Trail Making Test B; WBC: white blood cell count; YO: young-old adult

Table S2. Microbiome methodology of reviewed studies

Publication	Sequencing / Genetic Analysis	Alpha-Diversity Assessments	Beta-Diversity Assessments	Differential Abundance / Taxonomic Differences Methods	Functional Analysis Methods
Centenarian Studies					
Biagi et al., 2016	16S rRNA (V3-V4)<br>Illumina MiSeq (2x300 bp paired-end)	N/A	UniFrac (unweighted)	PCR amplification<br>Co-occurrence network analysis<br>Superimposed genus/family abundance on the PCoA plot to identify spatial correlations between samples and bacterial groups	N/A
Drago et al., 2012	16S rDNA<br>pyrosequencing	N/A	N/A	Colony bacteria counts (total aerobes, total anaerobes, *enterobacteriaceae*, *Enterococcus*, *Staphylococcus*, *Lactobacillus*, *Bifidobacterium*, *Clostridium*, *Bacteroides*, and yeast)<br>Pulsed field gel electrophoresis	N/A
Kim et al., 2019	16S rRNA (V1-V3) pyrosequencing	Observed OTUs<br>Shannon index	Bray-Curtis	Heatmap analyses<br>Random Forests<br>LEfSe	PICRUSt (KEGG orthology)
Kong et al., 2016	16S rRNA (V4-V5)<br>Illumina MiSeq (2x250 bp paired protocol)	Chao index,<br>Observed OTUs Shannon index	Bray-Curtis<br>Jaccard	Random forests	N/A
Rampelli et al., 2013	Shotgun metagenomic sequencing<br>Illumina (no further details)	Simpson index	Euclidean distance	MetaPhlAn	MetaCV pipeline (KEGG orthology)
Tuikhar et al., 2019	16S rRNA (V4-V5)<br>Illumina MiSeq<br>qPCR	Chao1<br>Shannon index	Bray-Curtis	qPCR (*Prevotellaceae)*<br>Random Forests	LC-MS
Wu et al., 2019	Shotgun metagenomic sequencing<br>Illumina HiSeqX10 PE150 platform (average insert size of 350 bp)	Observed OTUs Shannon index	Bray-Curtis	NMDS<br>MetaPhlan2.0	Humann2 (KEGG orthology)
Yu et al., 2015	16S rRNA (V4)<br>Illumina MiSeq (250bp/300bp paired-end)<br>qPCR	Chao1<br>Observed OTUs<br>Shannon index	UniFrac (unweighted)	qPCR (*Bacteroides, Bifidobacterium, Enterococcus, Enterobacteriaceae, Clostridium perfringens, Lactobacillus*)	N/A
Developmental Aging Studies					
Claesson et al. 2012	16S rRNA (V4)<br>454 Genome Sequencer FLX Titanium platform	Shannon Index<br>Observed OTUs<br>Phylogenetic diversity	UniFrac (weighted and un-weighted)<br>non-UniFrac (Bray-Curtis, Jaccard, Chisq, Chord, Euclidean, Hamming, Lennon, Ochiai, Pearson, Sorenson, Canberra, Hellinger, Manhattan,	PLS-DA	NMR spectroscopy

			Kulczynski, Morisita, Soergel)		
Hippe et al., 2011	16S rRNA genes and metabolic genes<br>qPCR	N/A	N/A	qPCR (*Clostridium* clusters IV and XIVa)<br>Melt curves analyses	Quantification by qPCR
Hopkins et al. 2002	16S rRNA	N/A	N/A	Viable count (total facultative anaerobes, total anaerobes, *bifidobacteria, enterococci, C. dificile, enterobacteria, Clostridium perjkzgens, lactobacilli, Bacteroides, Porphyromonas, Prevotella*)	CFA analysis
Jeffery et al., 2016	16S rRNA (V4)<br>454 Genome Sequencer FLX Titanium platform	Chao1<br>Shannon Index<br>Simpson<br>Phylogenetic diversity	UniFrac	N/A	N/A
Kato et al., 2017	16S rDNA<br>qPCR	N/A	N/A	qPCR (*B. adolescentis* group, *B. animalis* ssp. *Lactis, B. bifidum, B. breve, B. catenulatum group, B. dentium B. gallinarum group, B. longum group, B. longum ssp. longum, B. minimum, B. mongoliense)*<br>Melt curves analyses	N/A
Kushugulova et al., 2015	16S rDNA<br>3730 XL DNA Analyzer	N/A	N/A	Not reported	Not reported
Le Roy et al., 2015	16S 23S rRNA intergenic spacer region<br>qPCR	N/A	N/A	N/A	NMR-based metabonomics, O-PLS, model was derived from ^{1}H-NMR spectra of fecal waters
Odamaki et al., 2016	16S rRNA (V3-V4)<br>Illumina MiSeq<br>qPCR	Chao1<br>Observed OTUs<br>Shannon index<br>Phylogenetic distance whole tree	UniFrac (weighted and un-weighted)	LefSe<br>qPCR (*Bacteroides uniformis, Bifidobacterium longum, Blautia producta, Escherichia coli, Parabacteroides distasonis*)	PICRUSt (KEGG orthology)
Odamaki et al., 2018	16S rRNA (V3-V4)<br>Illumina MiSeq<br>Strain-specific PCR	N/A	N/A	Calculated detection rate of gene families<br>Strain-Specific PCR (*B. longum* subsp. *Longum)*	Comparative genomics (GF defined by Markov Cluster Algorithm)
Pan et al., 2016	16S rRNA (V2-V3)<br>Vector NTI software, Clustal X<br>PCR-DGGE	Shannon-Wiener (only for diversity of genus *Lactobacillus*)	N/A	DGGE fingerprinting	N/A
Ruiz-Ruiz et al., 2019	N/A	Margalef microbial richness<br>Pielou's evenness<br>Shannon index	N/A	N/A	LC-MS
Singh et al., 2019	16S rRNA (V1-V3)<br>Illumina MiSeq (2x300 bp)	Chao1<br>Shannon Index	Bray-Curtis	DESeq2 1.12.3	N/A
Cognition Studies					
Anderson et al., 2017	16S rRNA<br>454 FLX Titanium based strategy	N/A	N/A	Not reported	N/A

Manderino et al., 2017	16S rRNA<br>454 FLX Titanium based strategy.	N/A	N/A	Not reported	N/A
Verdi et al., 2018	16S rRNA<br>Illumina MiSeq (2x250 bp paired-end)	Chao1<br>Phylogenetic diversity<br>Observed OTUs<br>Shannon Index	N/A	Not reported	N/A
Intervention Studies					
An et al., 2015	16S rDNA (V5-V6)<br>Illumina HiSeq2500 (2×150 bp)	Faith's phylogenetic diversity<br>Inverse Simpson	Unifrac (weighted and unweighted)	Qubit dsDNA BR Assay Kit with Qubit Fluorometer	Gas chromatography coupled with a flame ionization detector
Björklund et al., 2011	qPCR, non-selective DNA-based method, percent guanine-plus cytosine (%G+C) profiling	N/A	N/A	qPCR (*Atopobium*, *Bacteroides–Prevotella–Porphyromonas* group, *Lactobacillus*, *B. coccoides–E. rectale* group, *Clostridium* cluster XIVab, *F. prausnitzii* and related strains)<br>G+C content regression analysis	N/A
Spaiser et al., 2015	16S rRNA<br>pyrosequencing<br>qPCR	Chao 1<br>Observed OTUs	UniFrac	qPCR (*Bifidobacteria,* lactic acid bacteria, *Escherichia coli*)	N/A
Valentini et al., 2014	16S rDNA gene-targeted qPCR ABI model 373A DNA sequencer	N/A	N/A	16S rDNA gene-targeted qPCR (*Clostridium* cluster IV, *bifidobacteria*)	N/A

Abbreviations Used: BLAST: Basic Local Alignment Search Tool; CFA: cellular fatty acid; DDGE: denaturing gradient gel electrophoresis; KEGG: Kyoto Encyclopedia of Genes and Genomes; LC-MS: liquid chromatography and mass spectrometry analysis; LEfSe: linear discriminant analysis coupled with effect size measurements; NMDS: nonmetric multidimensional scaling; NMR: nuclear magnetic resonance; O-PLS: orthogonal projection to latent structure; OTU: operational taxonomic unit; PCR: polymerase chain reaction; PCoA: principal coordinates analysis; PLS-DA: partial least squares regression; qPCR: quantitative polymerase chain reaction/real-time polymerase chain reaction; rDNA: ribosomal deoxyribonucleic acid; rRNA: ribosomal ribonucleic acid; V: variable region of 16S rRNA

Table S3A. Results of studies of the gut microbiome in long-lived individuals

Authors	Alpha-Diversity (α)	Beta diversity (β)	Taxonomic Composition	Functional Potential and Metabolites
Biagi et al., 2016	N/A	Different between all possible comparisons of age groups (SCTN, LL, YO, ADT), except between SCTN and CTN (unweighted UniFrac)	**Changes with aging (SCTN, LL, YO, ADT):**<br>Family: ↓ *Bacteroidaceae,* ↓ *Lachnospiraceae,* ↓ *Ruminococcaceae,* ↑ Synergistaceae, and ↑ Christensenellaceae with aging; *cumulative relative abundance, no inferential statistics provided<br><br>Genera: ↓ *Coprococcus,* ↓ *Roseburia,* ↓*Faecalibacterium,* ↓*Bacteroides,* ↑ *Oscillospira,* ↑ *Odoribacter,* ↑ *Butyricimonas,* ↑ *Eggerthella,* ↑ *Akkermansia,* ↑ *Anaerotruncus,* ↑ *Bilophila,* ↑ *Bifidobacterium* (↓ YO and LL, compared to ADT, and ↑ SCTN); *no inferential statistics provided	N/A
Drago et al., 2012	N/A	N/A	**CTN vs. ADT:**<br>Family/Genera: CTN ↓ *Enterobacteriaceae*, ↓ *Bifidobacteria*, ↓ *Bacteroides*<br><br>Species: CTN ↑ *Bifidobacterium longum*, ↑ *Clostridia sensu stricto*	N/A
Kim et al., 2019	**LL vs. YO vs. ADT:**<br>No difference across groups (Shannon, observed OTUs)<br><br>**Longevity villages vs. Urbanized towns:**<br>No difference between groups (Shannon, observed OTUs)<br><br>**LL: CM vs. RH:**<br>No difference between groups (Shannon, observed OTUs)	**LL vs. YO vs. ADT:**<br>Did not report<br><br>**Longevity villages vs. Urban towns:**<br>Different between groups in PCoA (Bray-Curtis); *no inferential statistics reported<br><br>**LL: CM vs. RH:**<br>Did not report	**LL vs. YO vs. ADT:**<br>Phylum: LL ↑ Verrucomicrobia, compared to YO, and ↑ Verrucomicrobia, ↑ Proteobacteria, ↑ Actinobacteria, compared to ADT; YO ↓ Bacteroidetes and ↑ Proteobacteria, compared to ADT<br><br>Family/Genera: LL ↓ *Faecalibacterium,* ↓ *Prevotella,* ↑ *Escherichia,* ↑ *Akkermansia,* ↑ *Clostridium,* ↑ *Collinsella,* ↑ *Streptococcus*, ↑ uncultured *Christensenellaceae*, compared to YO and ADT<br><br>**Longevity villages vs. Urban towns:**<br>Phyla: Longevity villages ↓ Bacteroidetes, ↑ Firmicutes, compared to urban towns<br><br>Family/Genera: Longevity villages ↓ uncultured *Lachnospiraceae*, ↑ *Intestinibacter,* ↑ *Romboutsia,* ↑ *Turicibacter,* ↑ *Eubacterium_g5,* ↑ *Blautia,* ↓ *Lachnospira,* ↓ *Roseburia,* ↓ *Bacteroides*, compared to urban towns; YO in longevity villages ↑ *Ruminococcus_g2*, compared to ADT in longevity villages and urban towns; longevity villages ↑ *Lactobacillus*, compared to YO/ADT in urban towns; LL ↓ *Dialister*, compared to ADT in urban towns<br><br>**LL: CM vs. RH:**<br>Phyla: Community-dwelling ↑ Firmicutes, ↑ Tenericutes ↓ Bacteroidetes, ↓ Proteobacteria, ↓ Actinobacteria, ↓ Verrucomicrobia; *proportions, no inferential statistics provided<br><br>Genera: Community-dwelling ↑ Faecalibacterium, ↑ Intestinibacter, ↑ Eubacterium_g23, ↑ Lactobacillus, ↑ unclassified Lacnospiraceae, ↑ Dialister, ↑ Blautia, ↑ Eubacterium_g5, ↑	**LL vs. YO vs. ADT:**<br>KEGG Level 1: LL and ADT ↑ pathways related to metabolism, compared to YO; ↓ pathways related to genetic information processing in LL, then YO, then ADT; LL and YO ↑ pathways related to environmental information processing, compared to ADT.<br><br>KEGG Level 3: 26 metabolic pathways different between groups; of these, LL ↑ phosphatidylinositol signaling system, compared to YO and ADT; LL and ADT ↑ glycosphingolipid biosynthesis, compared to YO, and LL ↑ N-glycan biosynthesis, compared to YO and ADT<br><br>**LL: CM vs. RH:**<br>KEGG Level 3: carbohydrate metabolism different between groups (direction not specified).

			Agathobacter, ↑ Holdemanella, ↑ Lachnospira, ↑ Roseburia, ↑ Alloprevotella, compared to rehabilitation hospital	
Kong et al., 2016	LL ↑ observed OTUs, ↑ Chao, ↑ Shannon, compared to ADT; findings validated an independent (Italian) cohort. In RF models, Chao and observed OTUs among top predictors distinguishing LL vs. ADT.	Did not assess between LL vs. ADT; different between Italian and Chinese LL cohorts (Bray-Curtis, Jaccard)	**LL vs. ADT:**<br>Family/Genera: LL ↑ *Ruminococcaceae,* ↑ *Christensenellaceae*, ↑ *Clostridium* cluster XIVa, ↑ *Akkermansia*	N/A
Rampelli et al., 2013	N/A	Different between LL and different from YO (Euclidean distance)	**LL vs. YO**<br>Genera: LL ↑ *Escherichia*, and ↑ *Ruminococcus*, compared to YO; YO ↑ *Faecalibacterium*, ↑ *Eubacterium*, and ↑ *Bifidobacterium*, compared to LL	**Diversity Findings for KOs:**<br>α: No differences between LL and YO for KEGG pathways (Simpson index)<br><br>β: LL different from YO and ADT for KEGG pathways (Euclidean distance)<br><br>**Differential ranking of KEGG pathways related to aging:**<br>Aging (LL, YO) associated with ↑ metabolism of aromatic amino acids (tryptophan and phenylalanine), metabolism of amino acids (tyrosine, valine and lysine); ADT profile associated with ↑ metabolism of amino acids (histidine) and carbohydrates (glucose, galactose), pyruvate, and butanoate, and ↑ SCFA production.
Tuikhar et al., 2019	LL ↑ Chao1, compared ADT (both in the same village and external groups); no difference in Shannon index<br><br>***Ruminococcaceae* family-specific α-diversity:** LL ↑ Chao1 and Shannon index, compared to ADT	Different between LL and ADT (Bray-Curtis)	**LL vs. ADT (combined datasets: Indian, Italian, Japanese and Chinese):**<br>Family/Genera: LL ↓ *Prevotellaceae*, ↑ *Eggerthella,* ↑ *Rikenellaceae*, ↑ *Alistipes*, ↑ *Porphyromonadaceae*, ↑ *Parabacteroides*, ↑ *Porphyromonas*, ↑ *Odoribacter*, ↑ *Butyricimonas*, ↑ *Alicyclobacillaceae*, ↑ *Alicyclobacillus*, ↑ *Clostridiaceae*, ↑ *Finegoldia*, ↓ *Ruminococcaceae*, ↓ *Faecalibacterium*, ↑ *Anaerotruncus*, ↑ *Enterobacteriaceae*, ↑ *Desulfovibrionaceae*, ↑ *Desulfovibrio*, ↑ *Synergistaceae*, ↑ *Pyramidobacter*, ↑ *Verrucomicrobiaceae,* ↑ *Akkermansia* and ↑ *Clostridiales Family XI Incertae Sedis*, compared to ADT<br><br>Species: LL ↑ *Alistipes shahii*, ↑ *Porphyromonas uenonis*, ↑ *Odoribacter splanchnicus*, ↑ *Parabacteroides goldsteinii*, ↑ *Alicyclobacillus acidoterrestris*, ↑ *Finegoldia magna*, ↑ *Clostridium aminobutyricum*, ↑ *Clostridium p_enrichment_culture_clone_7_25,* ↑ *Clostridium sp_Kas107_1*, ↑ *Clostridium hathewayi*, ↑ *Eubacterium siraeum*, ↑ *Clostridium cellulolyticum*, ↑ *Clostridium asparagiforme*, ↑ *Faecalibacterium prausnitzii*, ↑ *Clostridium methylpentosum*, ↑ *Anaerotruncus colihominis*, ↑ *Escherichia albertii*, ↑ *Pyramidobacter piscolens*, ↑ *Akkermansia muciniphila,* compared to ADT	109 out of 871 metabolites significantly different between LL and ADT.<br><br>**Diversity Findings for metabolites:**<br>β: LL different from ADT (Bray-Curtis)<br><br>**Differential abundance of metabolites in LL vs. ADT (Indian dataset):**<br>LL ↑ DL-3-Aminoisobutyric acid, ↑ N-Ethylglycine, ↑ gamma-Aminobutyric acid (GABA), ↑ Imidazoleacetic acid, ↑ Niridazole, ↑ Erucic acid, ↑ Dihydroxyphthalic acid, ↑ Nitridazole, ↑ Triacetin, ↑ Goralatide, compared to ADT internal and external; ↓ cyclohexanecarboxylic acid, compared to ADT internal; ↓ 13-cis,16-cis-Docosadienoic acid, compared to ADT external

			**LL vs. ADT (Indian dataset):**<br>Family/Genera: LL ↑ *Rikenellaceae,* ↑ *Alistipes,* ↑ *Holdemania,* ↑ *Fusobacteriaceae,* ↑ *Fusobacterium,* ↑ *Proteus,* ↓ *Prevotellaceae,* ↓ *Prevotella*, compared to ADT<br><br>Species: LL ↑ *Bacteroides caccae,* ↑ *Clostridium hiranonis,* ↑ *Clostridium sp_MLG480,* ↑ *Fusobacterium mortiferum,* ↑ *Clostridium bifermentans,* ↑ *Clostridium innocuum,* ↓ *Prevotella copri*, compared to ADT<br><br>**Indian vs. Other datasets (Italian, Japanese and Chinese):**<br>Family: Indian ↑ *Erysipelotrichaceae*, ↑ *Enterobacteriaceae*, ↑ *Lactobacillaceae*, ↑ *Leuconostocaceae*, ↑ *Actinomycetaceae*, ↑ *Corynebacteriaceae*, ↓ *Rikenellaceae*, ↓ *Bacteroidaceae*, ↓ *Porphyromonadaceae*, ↓ *Bifidobacteriaceae,* compared to other three countries	
Wu et al., 2019	No difference across age groups (LL, YO, ADT) (Shannon index, observed OTUs); "core microbiota" (present in >50% samples) of LL ↑ species richness, compared to YO and ADT	LL different from YO and ADT (Bray-Curtis)	**LL vs. YO vs. ADT:**<br>Phyla: LL ↑ Proteobacteria, compared to YO and ADT; LL ↓ Firmicutes and ↓ Firmicutes/Bacteroidetes ratio, compared to YO<br><br>Genera: LL ↓ *Faecalibacterium,* ↓ *Ruminococcus,* ↓ *Corprococcus,* ↓ *Dorea,* ↑ *Methanobrevibacter*, compared to YO and ADT<br><br>Species: LL ↓ *Faecalibacterium prausnitzi*, ↓ *Eubacterium rectale*, ↑ *Bifidobacterium adolescentis*, ↓ *Ruminococcus sp_5_1_39BFAA*, ↓ *Dorea longicatena*, ↑ *Methanobrevibacter smithii*, compared to YO and ADT	115 out of 463 gene pathways significantly different among age groups<br><br>**Diversity Findings for KOs:**<br>α: LL ↑ Shannon and ↑ observed KOs, compared to YO and ADT; no difference between YO and ADT<br><br>β: LL different from YO and ADT (Bray-Curtis); no difference between YO and ADT<br><br>**Differential abundance of KOs in LL vs. YO vs. ADT:**<br>LL ↑ pathways related to central metabolism (glycolysis, pentose phosphate pathways, and tricarboxylic acid cycle), ↑ anaerobic respiration, ↑ aerobic respiration, ↑ metabolism of and fermentation to SCFAs (propanoate and acetate), ↓ amino acid biosynthesis pathways (e.g., L-lysine-, L-isoleucine- and L-methionine), ↑ aromatic compounds (e.g., L-phenylalanine metabolism and chorismite biosynthesis), ↓ pathways related to carbohydrate degradation, ↑ vitamin B2 and K2 synthesis pathways, ↑ KOs related to phosphotransferase system, F420, and coenzyme M, compared to YO and ADT; LL and YO ↓ vitamin B1 synthesis pathways, compared to ADT
Yu et al., 2015	LL ↑ Chao1 and Shannon index, compared to CK; *only mean,	Different between CK and LL (unweighted UniFrac)	**LL vs. CK:**	N/A

	SD values reported; no inferential statistics provided		Phylum: LL ↓ Firmicutes, ↑ Bacteroidetes, ↑ Proteobacteria, ↑ Verrucomicrobia, ↑ Spirochaetes, ↑ Synergistetes, ↑ Thermi<br><br>Genera: ↑ *Escherichia*, ↑ *Phascolarctobacterium*, ↑ *Parabacteroides*, ↑ *Desulfovibrio*, ↑ *Syntrophomonas*, ↑ *Novosphingobium*, ↓ *Faecalibacterium*	

Abbreviations Used: ADT: adult; CK: control group; CM: community-dwelling older adults; CTN: centenarian group; KEGG: Kyoto Encyclopedia of Genes and Genomes; KO: Kyoto Encyclopedia of Genes and Genomes (KEGG) orthology; LL: long-living, oldest-old adults; OTU: operational taxonomic unit; PCoA: principal component analysis to reduce dimension of variables; RF: random forest; SCTN: semi-supercentenarians; RH: rehabilitation hospital older adults; SD: standard deviation; YO: young-old adult

Table S3B. Results of studies of the gut microbiome across the lifespan

Authors	Alpha-Diversity (α)	Beta diversity (β)	Taxonomic Composition	Functional Potential and Metabolites
Claesson et al. 2012	**YO (CM vs. DH vs. RH vs. LS):** CM ↑ microbial diversity (Number unique OTUs, Shannon index, Phylogenetic diversity) and health food diversity, compared LS; CM ↑ microbial diversity (Number unique OTUs, Shannon index, Phylogenetic diversity), compared to DH; CM ↑ microbial diversity (Number unique OTUs, Phylogenetic diversity) and health food diversity, compared to RH; RH ↑ microbial diversity (Number unique OTUs) and health food diversity, compared to LS<br><br>**Dietary groups: DG1 (low fat/high fiber) vs. DG2 (moderate fat/high fiber) vs. DG3 (moderate fat/low fiber) vs. G4 ('high fat/low fiber):** Participants with DG1 ↑ microbial diversity (Number unique OTUs, Shannon index, Phylogenetic diversity) and health food diversity, compared to DG2, DG3, DG4; DG2 ↑ microbial diversity (number unique OTUs, Phylogenetic diversity) and health food diversity, compared to DG3, DG4; DG2 ↑ microbial diversity (Number unique OTUs, Shannon index, Phylogenetic diversity, health food diversity), compared to DG3	Different between CM and LS (unweighted and weighted UniFrac); no difference between CM and ADT	**CM vs. DH vs. RH vs. LS:** Phyla: LS ↑ Bacteroidetes, compared to CM; CM ↑ Firmicutes, compared to LS<br><br>Family/Genera: CM ↑ *Coprococcus*, ↑ *Roseburia*, ↑ *Lachnospiraceae*, compared to LS; LS ↑ *Parabacteroides*, ↑ *Eubacterium*, ↑ *Anaerotruncus*, ↑ Lactonifactor, ↑ *Coprobacillus*, compared to CM<br><br>**YO vs. ADT** Genus: YO ↓ *Ruminococcus*, ↓ *Blautia*, ↑ *Escherichia/Shigella*, compared to ADT	**CM vs. RH vs. LS:** CM ↑ total predicted gene counts, compared to RH, LS<br><br>**Co-inertia analysis of the microbiota and metabolome:** CM and RH ↑ acetate, ↑ propionate, ↑ valerate, compared to LS<br><br>**CM vs. LS:** NMR identification of chemical shifts of metabolites: CM ↑ butyrate, ↑ glutarate, ↓ glycine, ↓ glucose, ↓ lipid, relative to LS<br><br>**Gene counts for enzymes:** CM ↑ butyrate, ↑ acetate, ↑ propionate, compared to LS; RH ↑ butyrate, ↑ acetate, compared to LS<br><br>**Average sequencing coverage for enzymes:** CM ↑ butyrate, ↑ acetate, ↑ propionate, compared to LS; RH ↑ butyrate, compared to LS
Hippe et al., 2011	N/A	N/A	**YO vs. ADT omnivores vs. ADT vegetarians:** Genus: YO ↓ *Clostridium cluster XIVa*, compared to ADT omnivores and ADT vegetarians	**YO vs. ADT omnivores vs. ADT vegetarians:** YO ↓ butyryl-CoA:acetate CoA-transferase gene, compared to ADT; ↑ ADT vegetarians, compared ADT omnivores.

			Species: (Melt curve analysis) YO ↓ *Eubacterium hallii* / *Anaerostipes coli*, ↓ *E. rectale*/*Roseburia* spp., ↓ *F. prausnitzii* melt peaks, compared to ADT omnivores and ADT vegetarians	
Hopkins et al. 2002	N/A	N/A	**Healthy (YO vs. ADT vs. CHD) vs. elderly *Clostridium difficile*-associated diarrhea (NHYO):**<br>Genus: NHYO ↓ *Bacteroides*, compared to CHD, ADT and YO; NHYO ↑ *lactobacillus*, ↑ *clostridia*, compared to ADT and YO; YO, NHYO ↓ *Bifidobacteria*, compared to CHD, ADT.<br><br>Family: ADT ↓ *Enterobacteria*, compared to CHD, NHYO	NHYO ↑ Saturated straight chain (20:0), ↑ Unsaturated straight chain (20:1 cis ll), ↓ Saturated straight chain (12:0, 15:0) and absence of the branched chain (15:O ante and 15:O iso fatty acids); ADT ↑ branched chain CFA, compared to all other groups; ↑ dimethyl acyl (18.1 cisl1 DMA, 14.0 DMA), compared to CHD and NHYO; ↑ 15:0 ante DMA, compared to other groups; CHD did not have dimethyl acyl (18:0 DMA), unsaturated straight chain (16:1 cis9), compared to other groups.
Jeffery et al., 2016	↑ stable-microbiota CM, compared to unstable* CM (Shannon diversity); ↑ LS, compared to unstable* LS (Shannon diversity, Chao 1, phylogenetic diversity); ↑ stable-CM, compared to LS (Shannon diversity, Phylogenetic diversity, Simpson diversity)<br><br>*Participants in highest quartile of absolute distance between time point T0 (baseline) and T3 (3 months) are denoted as unstable	**LS vs. CM:**<br>Different between LS and CM (unweighted UniFrac);<br><br>**DH vs. RH vs. LS vs. CM:**<br>DH and RH had microbiota clusters in between LS and CM (unweighted UniFrac)<br><br>**ADT vs. CM:**<br>No difference between ADT and CM (unweighted UniFrac)	N/A	N/A
Kato et al., 2017	N/A	N/A	**Pre-weaning to 100 plus years**<br>Species: *B. longum* detected in all groups; Elderly ↑ *B. dentium*, ↓ *B. catenulatum*; Adult ↑ *B. adolescentis*, ↓ *B. breve*, ↑ *B. gallinarum*, ↑ *B. catenulatum*; Infant ↑ *B. breve*, ↓ *B. adolescentis*	N/A
Kushugulova et al., 2015	N/A	N/A	**LL vs. YO vs. ADT**<br>Phylum: ADT ↑ Bacteroidetes; YO ↑ Firmicutes; LL ↑ Tenericutes, compared to other groups; *no inferential statistics provided<br><br>Species: LL ↓ butyrate-producing and mucin-degrading species, compared with YO, ADT; *no inferential statistics provided	N/A
Le Roy et al., 2015	N/A	N/A	**YO vs. ADT**<br>YO ↑ *L. paracasei*, ↑ *L. plantarum*, ↓ *L. salivarius*, and ↓ *L. helveticus*, compared to ADT	**Correlation between *Lactobacillus* and metabolites:**<br>↑ *Lactobacillus* sp. was associated with ↑ SCFAs: acetate, propionate and butyrate, ↑

				lactic acid, ↑ amino acid: (tyrosine, phenylalanine, leucine, isoleucine, valine and lysine); *L. helveticus* was associated with ↑ butyrate, ↑ lactate, ↑ glucose<br><br>No difference between ADT and YO in H-NMR metabolic profiles
Odamaki et al., 2016	↑ with age (Chao1, number of observed species, Shannon index, phylogenetic distance whole tree)	Variation in data due to age (UniFrac distances, both weighted and un-weighted analyses); no differences in gender (UniFrac distances, both weighted and un-weighted analyses)	**Composition across all ages (0 to 100+):**<br>Phyla: With ↑ Age, ↓ Actinobacteria, ↑ Bacteroidetes, ↑ Proteobacteria	**Infant/Elderly vs. Adult enriched clusters:**<br>Pre-weaned infants ↓ xylose transporter<br>Infant/Elderly ↑ drug transporters
Odamaki et al., 2018	N/A	N/A	**Across age groups (pre-weaning to 100+ age)**<br>Species: *Blautia wexlerae, Streptococcus salivarius, Bifidobacterium longum*; *no inferential statistics provided, detected >50% of participants across age groups	**Negative correlation between ORF and age:** ↓ Number of ORF of *B. longum* subsp. *longum* strains with ↑ in the age of participants<br><br>**Younger vs. Older (GF enriched in *B. longum* subsp. *longum* strains)**<br>Older (GF:11) ↓ GF involved in carbohydrate transport and metabolism, compared to infants (GF:22); Adults ↑ GF involved defense mechanisms, transcription and replication, recombination and repair, compared other groups.<br><br>169 GF enriched in *B. Longum* subsp. *longum* strains in younger participants vs. 55 GF enriched in older participants; younger participants ↑ sialidase-encoding cluster, ↑ an α arabinofuranosidase gene cluster, ↑ pNAC3 (a 10 kb plasmid) homologue, ↑ capsule biosynthesis related genes and a Type VII secretion system, ↑ some prophage regions found in the AH1206 episome; infants enriched in sialidase clusters; older ↑ extracellular α-L-arabinofuranosidases, putative multidrug-family ABC transporter (associated two-component system), a genetic cluster (Hsp20-family heat shock chaperone), ↑ prophage regions
Pan et al., 2016	No difference between YO Nanning and YO Bama subjects (only for diversity of genus *Lactobacillus*; Shannon-Wiener)	N/A	**Representative *Lactobacillus* species in YO**:<br>*W. confusa*, *L. mucosae*, *L. crispatus*, *L. salivarius*, and *L. delbrueckii*<br><br>**YO Bama vs. YO Nanning:**	N/A

			Species: Bama ↑ *W. confusa*, ↑ *L. salivarius*, ↓ *L. mucosae*, compared to Nanning<br><br>**Correlations with diet:**<br>Dietary fibers intake ↑ *W. confusa*, ↑ *L. salivarius*, ↓ *L. mucosae*, ↓ *L. crispatus*; fat intake ↓ *L. salivarius*, ↑ *L. mucosae*; protein intake ↑ *L. mucosae*; carbohydrate ↓ *L. mucosae*	
Ruiz-Ruiz et al., 2019	N/A	N/A	N/A	**Diversity Findings for metabolites:**<br>α: YO ↑ compared to CHD, ADT (microbial richness, Pielou's evenness, Shannon index)<br><br>**YO vs. ADT vs. CHD:**<br>YO ↓ tryptophan and indole production with ↑ age; YO ↓ TnaA, ↓ TrpB, ↓ tryptophan, ↓ indole, compared to CHD, ADT
Singh et al., 2019	No difference between YO and NHYO (Shannon, Chao1)	No difference between YO and NHYO (Bray-Curtis)	**YO vs. NHYO:**<br>Family/Genus: YO ↑ *Akkermansia*, ↑ *Erysipelotrichaceae UCG-003*, ↑ *Bacteroides*, ↓ *Streptococcus*, ↓ *Lactobacillus*, ↑ *Lachnospiraceae* (UCG-005)[1], ↓ *Escherichia*[1]/*Shigella*[1], ↑ *Cardiobacterium*, ↑ *Neisseria*, ↑ *Comamonas*, ↑ *Capnocytophaga*, ↓ *Bifidobacterium*, ↑ *Filifactor*, ↑ *Fusobacterium*, ↑ *Propionibacterium*, ↑ *Haemophilus*, ↑ *Corynebacterium*, ↓ *Rothia*, ↑ *Porphyromonas*, ↑ *Ruminococcaceae* UCG-014, ↑ *Prevotella* 2, ↑ *Peptoclostridium*, compared to NHYO; *no inferential statistics provided	N/A

Abbreviations Used: ADT: adult; CFA: cellular fatty acid; CHD: children; CM: community-dwelling older adults; DH: day-hospital older adults; DG: dietary group; DMA: dimethyl acid; GF: gene family; LL: long-living, oldest-old adults; LS: long-stay older adults; NHYO: non-healthy younger-old adult; NMR: nuclear magnetic resonance; ORF: open reading frame; OTU: operational taxonomic unit; RH: rehabilitation hospital older adults; SCFA: short chain fatty acid; TnaA; tryptophanase; TrpB: tryptophan synthase; YO: young-old adult

Table S3C. Results of studies investigating the relationship of the gut microbiome to cognition in older adults

Authors	Alpha-Diversity (α)	Beta diversity (β)	Taxonomic Composition	Functional Potential and Metabolites
Anderson et al., 2017	N/A	N/A	**Associations with cognition and sleep:**<br>Verrucomicrobia and Lentisphaerae: ↑ sleep quality.<br><br>Verrucomicrobia: ↑ word reading, processing speed<br><br>Lentisphaerae: ↑ cognitive flexibility; non-significant after accounting for sleep	N/A
Manderino et al., 2017	N/A	N/A	**NHYO vs. YO:**<br>YO ↓ Bacteroidetes, ↓ Proteobacteria, ↑ Firmicutes, ↑ Verrucomicrobia, compared to NHYO<br><br>**Associations with cognition:**<br>Phylum: ↑ Verrucomicrobia showed ↑ verbal learning, ↑ visual scanning, ↑ cognitive set-shifting, ↑ cognitive flexibility (word reading), ↑ cognitive flexibility (color naming); ↑ Firmicutes showed ↑ spatial perception and visual memory ↑ memory; ↑ Bacteroidetes correlated to ↓ spatial perception and visual memory, ↓ memory; ↑ Proteobacteria correlated to ↓ verbal Recognition/Discrimination, ↓ FAB, ↓ FAS	N/A
Verdi et al., 2018	**Associations with cognition:**<br>↑ Chao1, phylogenetic diversity, and observed OTU associated with ↓ reaction time and ↓ verbal fluency; the latter was no longer significant after accounting for frailty	N/A	**Associations with cognition:**<br>Order: ↑ Burkholderiales associated with ↓ reaction time<br>Class: ↑ Betaproteobacteria associated with ↓ reaction time	N/A

Abbreviations Used: FAB: Frontal Assessment Battery; FAS: Verbal Association Fluency; NHYO: non-healthy younger-old adult; OTU: operational taxonomic unit; YO: young-old adult

Table S3D: Results of studies investigating interventions targeting the microbiome in older adults

Authors	Alpha-Diversity (α)	Beta diversity (β)	Taxonomic Composition	Functional Potential and Metabolites
An et al., 2019	**YO vs. ADT (Baseline):**<br>No difference between ADT and YO at baseline (inverse Simpson's index and phylogenetic diversity)<br><br>**Pre- vs. Post-Pectin Supplementation):**<br>No difference (Faith's PD, inverse Simpson) in both ADT and YO	**YO vs. ADT (Baseline):**<br>Difference between ADT and YO (unweighted UniFrac); no significance difference between ADT and YO (weighted UniFrac)<br><br>**Placebo vs. Pectin Supplementation (Baseline):**<br>No significant difference (weighted and unweighted UniFrac)<br><br>**Pre- vs. Post-Pectin Supplementation:**<br>No significant difference (weighted and unweighted UniFrac). Smaller intra-individual (within person) change, compared to Inter-individual (across people) (weighted UniFrac and unweighted UniFrac)	**YO vs. ADT (Baseline):**<br>YO ↑ *Enterorhabdus*, ↑ *Ruminiclostridium* 6, ↑ uncultured genus within the family Coriobacteriaceae, ↑ *Mogibacterium*, ↑ *Lachnospiraceae* (UCG-008), compared to ADT<br><br>**YO vs. ADT (Post Pectin Supplementation):**<br>YO ↑ *Enterorhabdus*, ↑ uncultured genus within the family *Coriobacteriaceae*, ↑ *Mogibacterium*, ↑ *Lachnospiraceae* UCG-008)<br><br>**Pre- vs. Post-Pectin Supplementation:**<br>No change in taxonomic composition.	**YO vs. ADT (Baseline):**<br>No significant differences in BCFA or SCFA<br><br>**Pre- vs. Post-Pectin Supplementation:**<br>No significant differences in BCFA or SCFA (acetic acid, propionic acid, butyric acid, valeric acid, isobutyric acid, isovaleric acid) in YO or ADT
Björklund et al., 2011	N/A	N/A	**Placebo vs. Synbiotic:**<br>Genera: Synbiotic ↑ B*ifidobacterium*, ↑ *L. acidophilus NCFM,* compared to placebo; both (synbiotic and placebo) ↓ *Clostridium* cluster XIVab, ↓ *Blautia coccoides– Eubacterium rectale*	N/A
Spaiser et al., 2015	**Placebo vs. Probiotic:**<br>No difference between placebo and probiotic groups (Chao1, observed OTUs)	**Placebo vs. Probiotic:**<br>No difference between placebo and probiotic groups (UniFrac)	**Placebo vs. Probiotic:**<br>Genus: Probiotic ↑ *Bifidobacteria* and ↑ lactic acid bacteria, compared to placebo<br>Species: Probiotic ↓ *Escherichia coli* and ↑ *Faecalibacterium prausnitzii*, compared to placebo	N/A
Valentini et al., 2014	N/A	N/A	**Diet only vs. Diet+VSL#3 treatment (Low-grade Inflammation Group):**<br>Diet+VSL#3 ↑ *Bifidobacterium*<br><br>**Pre- and Post-Diet+VSL#3 treatment:**<br>No change in *Clostridium* cluster IV, *Bifidobacterium*	N/A

			spp.	

Abbreviations Used: ADT: adult; BCFA: branched chain fatty acids; FMT: fecal microbiota transplantation; OTU: operational taxonomic unit; PD: phylogenetic diversity; SCFA: short chain fatty acids; YO: young-old adult
